# Ferroptosis-driven radio-immunosensitization: an emerging mechanistic framework for enhancing response to immune checkpoint inhibitor therapy

**DOI:** 10.3389/fonc.2026.1954104

**Published:** 2026-10-08

**Authors:** Zhenghao Cao, Xudong Ao

**Affiliations:** 1 Department of Tumor Biological Therapy, Affiliated Cancer Hospital of Inner Mongolia Medical University, Hohhot, China; 2 Department of Radiation Oncology, Hohhot First Hospital, Hohhot, China; 3 Department of Tumor Biological Therapy, Peking University Cancer Hospital Inner Mongolia Hospital, Hohhot, China

**Keywords:** DHODH, ferroptosis, immune checkpoint inhibitors, radio-immunosensitization, radiotherapy, SLC7A11, treatment resistance

## Abstract

Immune checkpoint inhibitors (ICIs) provide durable tumor control in some patients, but resistance can develop through tumor-intrinsic and microenvironmental mechanisms. This narrative review examines the metabolic and immune conditions under which ferroptosis may enhance ICI responsiveness or resensitize selected ICI-refractory tumors. Radiotherapy (RT) increases oxidizable phospholipids and can repress SLC7A11, the light-chain component of system xc^-^. ICI-activated CD8^+^ T cells provide convergent interferon-γ signaling. Tumors can resist this pressure through GPX4, FSP1-CoQ10, mitochondrial DHODH, NRF2-dependent programs, and iron-export pathways. Recent preclinical studies have tested RT with ferroptosis-directed interventions and ICIs. These studies include functional evidence of ferroptosis dependence in radioresistant lung cancer and resensitization of LKB1-deficient, primarily ICI-refractory tumors. The findings support a preclinical rationale for combination therapy, although their causal strength varies. Reversal of clinically defined acquired ICI resistance remains unestablished, and the clinical RT-ICI studies reviewed here did not measure ferroptosis in patients. The immune effects also vary by cell type. Tumor-cell ferroptosis may support immunity, whereas lipid peroxidation in CD8^+^ T cells, dendritic cells, or polymorphonuclear myeloid-derived suppressor cells can impair antitumor responses. Translation will require functional validation, cell-resolved biomarkers, achievable drug exposures, and evaluation of RT dose, fractionation, and sequence alongside normal-tissue and immune-cell safety.

## 1 Introduction

Immune checkpoint inhibitors (ICIs) targeting PD-1, PD-L1, and CTLA-4 have changed the treatment of several malignancies. Durable benefit, however, remains limited to a subset of patients. Primary resistance may arise from low antigenicity, defective antigen processing or presentation, or exclusion of effector T cells. Tumor-intrinsic oncogenic programs, impaired interferon signaling, suppressive myeloid or stromal compartments, and systemic host factors can also contribute. Adaptive resistance can develop during treatment through checkpoint re-expression and compensatory immune suppression. Acquired resistance follows an initial response and may involve immune editing, loss of target antigens, JAK1/2 alterations, or renewed T-cell dysfunction (1–7). Ferroptosis evasion may contribute to ICI failure within particular biological contexts, alongside these other resistance mechanisms.

Radiotherapy (RT) is a plausible partner for ICI therapy because its effects extend beyond local DNA damage. Irradiation can activate cGAS-STING-dependent type I interferon signaling, promote antigen release and cross-presentation, and alter vascular and stromal barriers. These changes may support immune responses outside the irradiated field (8, 9, 11). PACIFIC, PEMBRO-RT, pooled pembrolizumab analyses, and studies of RT with CTLA-4 blockade have examined clinical activity across distinct treatment settings (12–17). Their results provide a clinical basis for studying RT with ICI therapy. The contribution of ferroptosis to these clinical outcomes requires separate mechanistic investigation.

Ferroptosis is an iron-dependent form of regulated cell death driven by phospholipid peroxidation (18–20). RT can increase lipid oxidation and suppress SLC7A11 through ATM-dependent signaling. ICI-activated CD8^+^ T cells release interferon-γ (IFN-γ), which can also repress SLC7A11 in tumor cells. These signals reduce cystine uptake and glutathione availability, weakening GPX4-mediated detoxification (21, 22). SLC7A11 forms the light chain of system xc^-^, the heterodimeric cystine/glutamate antiporter. The outcome of this convergence depends on tumor genotype, cell identity, tissue, timing, and other antioxidant defenses. This review examines how these factors shape ferroptosis-dependent radioimmunosensitization and the conditions required for its clinical investigation.

## 2 Review scope and approach

This article is a mechanistic narrative review. Publications were assembled around ferroptosis, radiation, immune-checkpoint blockade, and treatment resistance. Targeted checks during revision used PubMed records, Crossref/OpenAlex bibliographic metadata, and publisher sources. Search concepts included ferroptosis, radiotherapy, radiation, immune checkpoint, PD-1, PD-L1, resistance, SLC7A11, GPX4, FSP1, DHODH, HSPB1, nanomedicine, and myeloid-derived suppressor cell. Original experiments received priority for mechanistic claims, while clinical studies informed the assessment of treatment efficacy and feasibility. Extraction covered models, treatment schedules, endpoints, and rescue experiments where these details were available. Study-level reporting and validation limitations were distinguished from efficacy outcomes. The review used no prospectively registered protocol and makes no claim to exhaustive retrieval. Formal risk-of-bias scoring and meta-analysis were outside its scope.

Recent reviews describe the biological and therapeutic foundations of ferroptosis in cancer (23, 24). Lei et al. discuss ferroptosis-mediated tumor suppression, the tumor microenvironment, and therapeutic interventions across cancer settings (25). Wei et al. examine the intersection of ferroptosis, RT, and immunotherapy, including ATM/IFN-γ convergence and fractionation (26). Wang et al. focus on mitochondrial dysregulation and its interaction with ferroptosis during radioimmunotherapy (27). The present review combines an updated evidence base with a study-level assessment of radioimmunosensitization. It incorporates original studies published from 2024 to 2026, including recent nanomedicine, DHODH-targeting, and LKB1-related research. The assessment distinguishes mechanistic plausibility from functional ferroptosis validation and examines preclinical combination efficacy, resensitization in defined ICI-refractory models, and clinical benefit from RT with ICI. Cell-specific immune effects are considered alongside drug exposure and biomarker requirements. This focus connects recent mechanistic advances with the experimental and translational evidence needed to evaluate ferroptosis-directed combinations.

## 3 Ferroptosis machinery, validation, and immunological context

Ferroptosis occurs when iron-dependent oxidation of polyunsaturated-fatty-acid-containing phospholipids exceeds cellular repair capacity. ACSL4 and LPCAT3 shape the pool of oxidizable membrane phospholipids. Lipoxygenases and cytochrome P450 oxidoreductase can promote oxidation. Iron supplied through uptake, storage, and ferritinophagy supports radical propagation (30–38). Tumor suppressors such as p53 and BAP1 can lower SLC7A11 expression, while NRF2-dependent programs can reinforce antioxidant capacity (39–43).

Primary studies show how lipid substrates contribute to ferroptosis susceptibility. Genetic screens found that ACSL4 loss protected cells even in the absence of GPX4. Lipidomic analysis linked this protection to depletion of oxidizable polyunsaturated membrane lipids (31). Kagan et al. identified oxidized arachidonoyl- and adrenoyl-phosphatidylethanolamines as death-associated lipid species, connecting specific substrates to ferroptosis execution (33). These measurements distinguish the relevant lipid injury from a general increase in ROS. NCOA4-mediated transport of ferritin to lysosomes provides another route for mobilizing stored iron (37). Differences in lipid composition and iron availability may therefore produce different ferroptotic thresholds despite similar GPX4 expression. **Table 1** summarizes these regulators and their relevance to translation.

**Table 1:** Ferroptosis regulators at the RT-ICI interface.

Regulator/pathway	Mechanistic role	Role in RT	Immune/ICI relevance	Translational interpretation
System xc^-^ (SLC7A11 light chain + SLC3A2 heavy chain)	Cystine uptake supports GSH synthesis and GPX4 activity	ATM-dependent SLC7A11 repression can lower the ferroptotic threshold	IFN-γ/STAT1 from activated CD8^+^ T cells can repress SLC7A11	SLC7A11 is one transporter component; its predictive clinical value requires validation
GPX4	Reduces phospholipid hydroperoxides using GSH	Opposes radiation-induced lipid peroxidation	Tumor inhibition may enhance killing; direct inhibition can endanger immune/normal cells	Direct inhibitors remain mainly research tools
ACSL4/LPCAT3	Creates PUFA-phospholipid substrates	Shapes RT-induced lipid-peroxidation susceptibility	Tumor-cell ACSL4 can support CD8^+^ T-cell-driven ferroptosis	Susceptibility marker; normal-tissue injury is a concern
FSP1-CoQ10	GSH-independent membrane antioxidant defense	Potential bypass when GPX4/system xc^-^ is suppressed	No validated clinical ICI-predictive role	Preclinical inhibitors; exposure and selectivity unresolved
DHODH	Mitochondrial ubiquinol production and pyrimidine synthesis	RT-induced DHODH supports acquired radioresistance and DNA repair	Inhibition cooperates with IFN-γ/anti-PD-1 to intensify RT-induced ferroptosis	Direct preclinical RT plus DHODH inhibition and anti-PD-1 evidence with Lip-1 rescue in acquired radioresistance
NRF2/HO-1	Adaptive antioxidant and iron-handling program	Can reinforce SLC7A11/GPX4 defenses; context-dependent	May define redox-resistant tumors	Broad pathway targeting risks off-target toxicity
PROM2	Ferritin-containing vesicle/exosome export reduces labile iron	Potential adaptive radioresistance mechanism	Indirect relevance to ICI response	Preclinical target; no clinical agent
HSPB1	Cytoskeletal/redox stress response linked to ferroptosis resistance	Silencing increased RT-associated ferroptosis in HCC models	Exploratory RT-anti-PD-1 evidence	Lower-certainty evidence; rescue and model validation incomplete
CD36 in CD8^+^ T cells	Oxidized-lipid uptake promotes lipid peroxidation and dysfunction	May increase immune-cell vulnerability after RT	Can blunt ICI efficacy	Potential immune-cell safety marker; tumor targeting requires a separate rationale
PMN-MDSC/neutrophil ferroptosis	Can generate immunosuppressive lipid signals	RT can alter myeloid trafficking and redox state	May weaken ICI response; targeted myeloid engineering is distinct from global induction	Requires cell-selective strategies

RT, radiotherapy; ICI, immune checkpoint inhibitor; GSH, glutathione; GPX4, glutathione peroxidase 4; IFN-γ, interferon-γ; CoQ10, coenzyme Q10; DHODH, dihydroorotate dehydrogenase; PMN-MDSC, polymorphonuclear myeloid-derived suppressor cell. Translational interpretations distinguish mechanistic relevance from clinically validated predictive utility.

Cystine uptake, glutathione synthesis, and GPX4 activity form the canonical defense pathway. System xc^-^ is a heterodimer formed by the SLC7A11 light chain and the SLC3A2 heavy chain. Imported cystine supports glutathione synthesis, and GPX4 reduces phospholipid hydroperoxides. Parallel safeguards include FSP1-dependent regeneration of reduced CoQ10 at cellular membranes, GCH1/BH4-dependent lipid remodeling, and mitochondrial DHODH-mediated ubiquinol production (45–48). PROM2-associated export of ferritin-containing vesicles can reduce the labile iron pool (49). These partially redundant defenses make susceptibility dependent on more than SLC7A11 or GPX4 expression alone.

The cellular location of antioxidant defenses influences their protective effects. Bersuker et al. identified FSP1 in a synthetic-lethal screen and showed that membrane recruitment sustains a CoQ antioxidant system parallel to GPX4 (44). DHODH supplies mitochondrial protection linked to pyrimidine metabolism, while PROM2 limits iron availability through export of ferritin-containing vesicles (48, 49). These defenses may preserve survival after inhibition of cystine transport. A tumor that survives SLC7A11 suppression could therefore retain an alternative protective pathway, even when ferroptotic stress contributes to its treatment response. Such a pathway may offer a target for a second intervention. **Figure 1** depicts these defenses, whose relative contributions vary among tumors.

**Figure 1:**
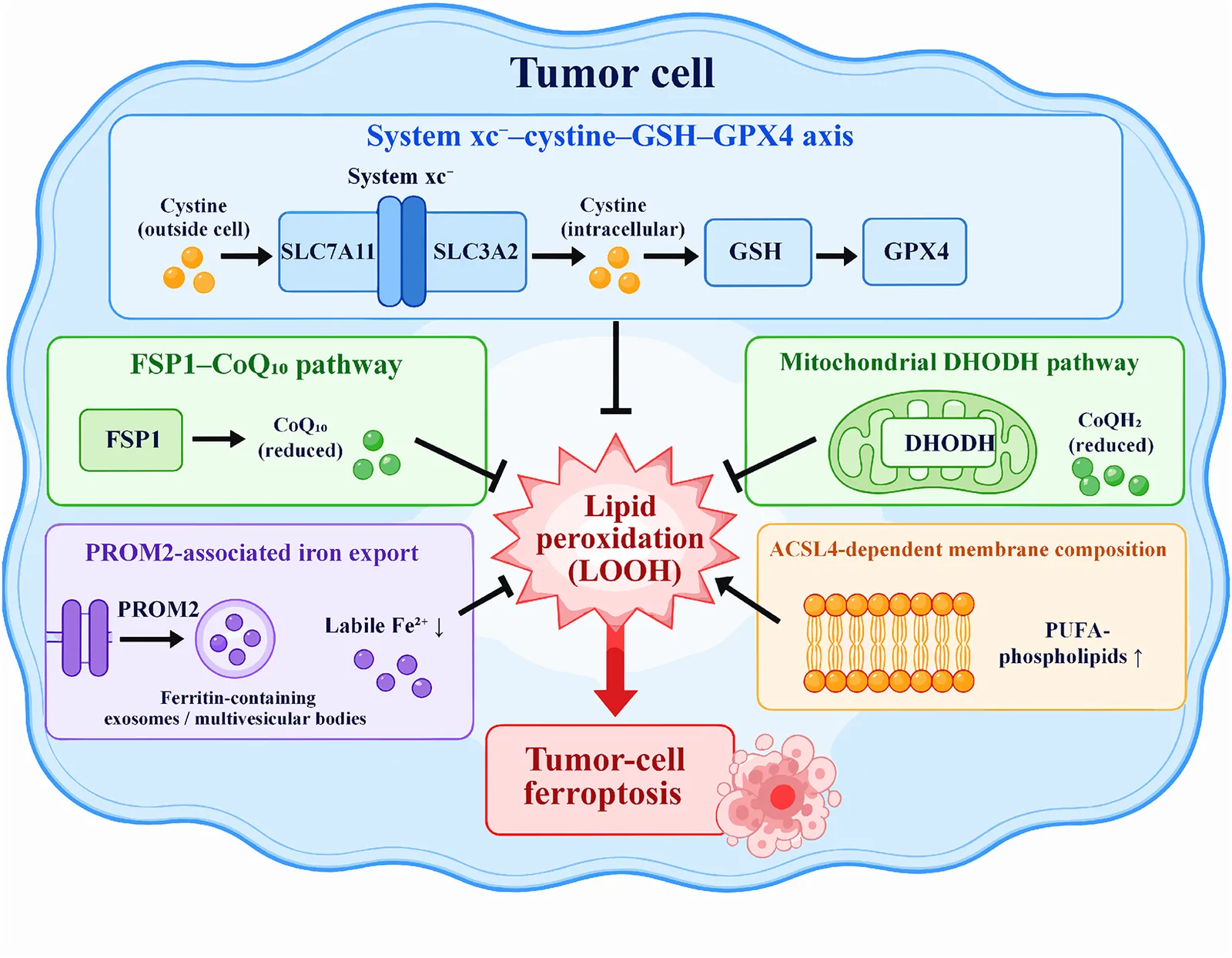
Determinants of tumor-cell ferroptosis susceptibility and resistance. Tumor-cell ferroptosis depends on the balance between lipid-peroxidative pressure and partially redundant defense pathways. System xc^-^ comprises the SLC7A11 light chain and SLC3A2 heavy chain. Its import of cystine supports glutathione (GSH) synthesis and GPX4-mediated detoxification of phospholipid hydroperoxides. FSP1 regenerates membrane-associated reduced coenzyme Q10 (CoQ10), while mitochondrial DHODH produces ubiquinol. Both mechanisms suppress lipid peroxidation independently of the canonical GPX4 pathway. PROM2-associated export of ferritin-containing vesicles can reduce the labile iron pool and limit iron-dependent oxidation. ACSL4 increases the availability of oxidizable membrane substrates through enrichment of polyunsaturated-fatty-acid-containing phospholipids (PUFA-PLs). Ferroptosis occurs when treatment-induced lipid injury exceeds the combined protection afforded by these pathways in a given tumor.

### 3.1 Minimum criteria for assigning ferroptosis

Ferroptosis assignment requires concordant biochemical and functional evidence. Phospholipid peroxidation can be assessed through C11-BODIPY oxidation or oxidized phospholipid profiling, with 4-HNE and malondialdehyde providing supporting oxidative-injury readouts. Measurements of labile Fe²^+^, glutathione depletion, or GPX4-system perturbation help characterize iron dependence and redox disruption. Functional testing should include rescue with ferrostatin-1 or liproxstatin-1 and, where feasible, a concordant genetic intervention. Iron chelation provides additional evidence of iron dependence, although it is less specific than ferroptosis-selective rescue. Electron microscopy and changes in protein expression can support the assignment when interpreted alongside rescue and independent biochemical readouts (20, 28, 29). Morphology, a single oxidative marker, or reduced GPX4 abundance alone provides insufficient evidence. Combination studies are most informative when they test rescue within the full regimen as well as after individual drug exposure.

RT experiments benefit from assessment of both ferroptosis rescue and durable reproductive survival. Short-term metabolic viability assays can register growth arrest, whereas clonogenic assays measure whether irradiated cells retain the capacity to form colonies. General ROS probes lack specificity for membrane phospholipid oxidation. Both 4-HNE and malondialdehyde can also arise during oxidative injury unrelated to ferroptosis. C11-BODIPY measurements and lipidomics add biochemical detail to functional tests. Radical-trapping inhibitors have antioxidant actions, so agreement between pharmacological and genetic rescue gives stronger support than a single inhibitor. Controls for apoptosis and necroptosis help distinguish mixed death responses (28, 29, 65). Ferroptosis still requires positive evidence when those controls are absent.

These criteria provide a basis for comparing causal evidence. Rescue within a regimen combining RT, a ferroptosis-directed intervention, and ICI gives stronger support than concurrent tumor control and lipid-peroxidation markers. Multifunctional nanomaterials require particular care in interpretation. Antigen capture, adjuvant delivery, metabolic changes, and ROS generation may each contribute to their effects. Factorial controls can help determine how much of the antitumor response depends on ferroptosis. **Table 2** applies this distinction to the preclinical studies discussed below.

**Table 2:** Direct RT-ferroptosis induction studies and representative preclinical RT-ICI evidence.

Study	Model	RT schedule and treatment sequence	Ferroptosis-directed intervention/ICI	Ferroptosis validation	Key efficacy/immune endpoints
Lei et al., 2020 (65)	A549 xenografts and KEAP1-mutant lung cancer PDX; immunodeficient hosts; no ICI arm	Xenograft: 10 Gy × 1; sulfasalazine 250 mg/kg i.p. daily, starting with 3 doses before RT. PDX: 8 Gy × 1; same pretreatment, then every 2 days	Sulfasalazine (system xc^-^ inhibition); cell assays also tested erastin, RSL3, FIN56 and ML162	Lipid ROS/4-HNE; ACSL4 genetic experiments and Fer-1/Lip-1 rescue support radiation-induced ferroptosis; not an RT-ICI rescue study	Clonogenic radiosensitization; xenograft/PDX growth inhibition; no intact-host ICI efficacy inference
Lang et al., 2019 (22)	Immunocompetent B16F10 and ID8 models; ICI-resistant state not established	8 Gy × 1 in B16F10; RT with anti-CTLA-4 or anti-PD-L1	No therapeutic FIN; Lip-1 used as mechanistic rescue; Anti-CTLA-4/anti-PD-L1	Tumor lipid ROS; Lip-1 weakens combined tumor control	Tumor control with RT + anti-CTLA-4/anti-PD-L1.
Song et al., 2024 (79)	A549 mechanistic/xenograft studies; LLC/C57BL/6 immunocompetent combination model; no established ICI-resistant state	PDPCA@ATRA 5 mg/kg i.v.; 24 h later local 10 Gy × 1; anti-PD-1–10 mg/kg i.p. on days 3, 6, 9, 12, and 15	PDPCA@ATRA glutathione-depleting nanomedicine; Anti-PD-1	C11-BODIPY, PTGS2, GSH/GSSG and Fer-1 rescue in component experiments; 4-HNE in A549 xenografts; no rescue within the LLC full triad	Tumor control; enhanced cytotoxic T-cell responses.
Xu et al., 2024 (80)	Bilateral 4T1-Luc breast tumors in BALB/c mice; ICI-resistant state not established	Local RT: 6 Gy × 2 on days 8 and 9 (1 Gy/min). Intratumoral FMC: days 9, 11, 13, starting after RT; intratumoral anti-PD-1: days 9, 12, 15. Day 0 = first tumor inoculation	CpG-loaded Fe_3_O_4_ antigen-capturing nanoadjuvant; PD-1 blockade	Metabolomic changes interpreted as consistent with ferroptosis; no ferroptosis-specific rescue reported in the main article	Control of irradiated and distant tumors; reduced lung metastasis; enhanced antitumor immune responses.
Zhu et al., 2024 (64)	4T1 and CT26 syngeneic BALB/c models; no established ICI-resistant state	Local 6 Gy × 2 on consecutive days; 72 h after the last RT, Fe_3_O_4_-anti-PD-L1 was given i.v. every 3 days × 3	PD-L1-targeted Fe_3_O_4_; Fenton chemistry and MDSC lipid peroxidation; Anti-PD-L1 is part of the targeting construct	C11-BODIPY, MDA, GPX4/ACSL4 and mitochondrial changes; DFO attenuated MDSC depletion and tumor control	MDSC depletion/remodeling; tumor control.
Luo et al., 2025 (71)	LLC/C57BL/6 immunocompetent model; six NSCLC patients with progression after prior platinum-doublet plus sintilimab	*In vivo* 1 Gy × 5; exploratory clinical 1.2 Gy × 5; anti-PD-1/ICI combined	No independent FIN; Anti-PD-1/sintilimab	C11-BODIPY, ROS, MDA, Fe²^+^, GSH and pathway markers; Fer-1 rescue in experimental systems; patient-level ferroptosis was not measured	Tumor control in experimental models; exploratory activity in six NSCLC patients.
Dou et al., 2026 (81)	Hepa1–6 HCC model; ICI-resistant state not established	8 Gy × 3; camrelizumab every 3 days; HSPB1 silencing	si-Hspb1; Anti-PD-1 (camrelizumab)	ROS, Fe²^+^, GPX4/ACSL4/SLC7A11 and morphology; no ferroptosis-selective rescue within the full regimen	Tumor control with HSPB1 silencing + RT + anti-PD-1.
Zhang et al., 2026 (82)	LLC1-sgLkb1 syngeneic C57BL/6 tumors; genetically defined LKB1-deficient primary ICI-refractory context	Local 8 Gy × 3; anti-PD-1 200 μg/mouse i.p. on days 1, 4, and 7; Lip-1–10 mg/kg on the same schedule for rescue	RT disrupts DNMT1-OSGIN1 ferroptosis tolerance; no separate FIN; Anti-PD-1	Tumor lipid ROS plus *in vivo* Lip-1 reversal of RT-ICI tumor control; RT concurrently restored STING-associated signaling	Suppression of LKB1-deficient tumors; context-specific ICI resensitization.
Mao et al., 2026 (70)	Acquired radioresistant 393P lung tumors; acquired ICI-resistant state not established	10 Gy × 1, then days 3/6/9 anti-PD-1 + DHODH inhibitor	Leflunomide 10 mg/kg or BAY-2402234–4 mg/kg; Dhodh deletion; Anti-PD-1, 100 μg/mouse	4-HNE/lipid peroxidation; Lip-1 rescue *in vitro* and *in vivo*; genetic confirmation	Tumor control in acquired radioresistant 393P tumors.

FIN, ferroptosis-inducing intervention; HCC, hepatocellular carcinoma; ICI, immune checkpoint inhibitor; Lip-1, liproxstatin-1; RT, radiotherapy. Resistance state is retained within the Model column where relevant because acquired radioresistance and acquired ICI resistance are not interchangeable.

### 3.2 Cell-type-specific immune effects of ferroptosis

Early ferroptotic cancer cells supported antitumor vaccination under defined experimental conditions (50, 51). Antigen release and damage-associated molecular-pattern signaling are established features of immunogenic cell death (52–55), although their expression varies during ferroptosis. Other experiments found impaired dendritic-cell maturation and cross-presentation after exposure to ferroptotic cancer cells, including cells in the initial phase of death (56). These divergent outcomes indicate that death stage, cargo composition, uptake pathways, and the local inflammatory environment all warrant attention when interpreting immune effects.

Oxidative pressure can also damage immune cells. Lipid peroxidation increases ferroptotic vulnerability or dysfunction in activated T cells, and CD36-dependent uptake of oxidized lipids suppresses intratumoral CD8^+^ T-cell effector activity (57, 59, 60). Ferroptotic tumor neutrophils and polymorphonuclear myeloid-derived suppressor cells (PMN-MDSCs) can acquire immunosuppressive properties (61, 62). A strategy combining tumor ferroptosis induction with MDSC blockade sensitized liver tumors and metastases to ICI therapy (63). Zhu et al. used an RT-guided Fe3O4-anti-PD-L1 nanoprobe to target MDSCs, induce lipid peroxidation, and alter the myeloid compartment (64). This study informs cell-selective treatment design and microenvironmental remodeling. Its implications for reversal of established ICI resistance require further testing.

The vaccination studies differ in several experimental features. Efimova et al. used RSL3-treated MCA205 and GL261 cells and found that early ferroptotic cells stimulated dendritic-cell maturation. Vaccination protected immunocompetent mice, with no corresponding protection in Rag-2-deficient mice (50). Wiernicki et al. used a temporally resolved inducible system. Initial ferroptotic cells were poorly engulfed, reduced antigen cross-presentation, and compromised vaccination, including when mixed with immunogenic apoptotic cells (56). An early or late classification therefore gives an incomplete account of immunogenicity. The induction method, residual viable cells, antigen processing, and recipient dendritic cells may influence the result. These features deserve consideration when extrapolating vaccination experiments to an irradiated tumor.

Selective tumor-cell ferroptosis is a more useful therapeutic objective than an overall increase in tissue lipid oxidation. A combination must also preserve CD8^+^ T-cell and dendritic-cell function or redirect suppressive myeloid populations. **Figure 2** summarizes these cell-specific effects.

**Figure 2:**
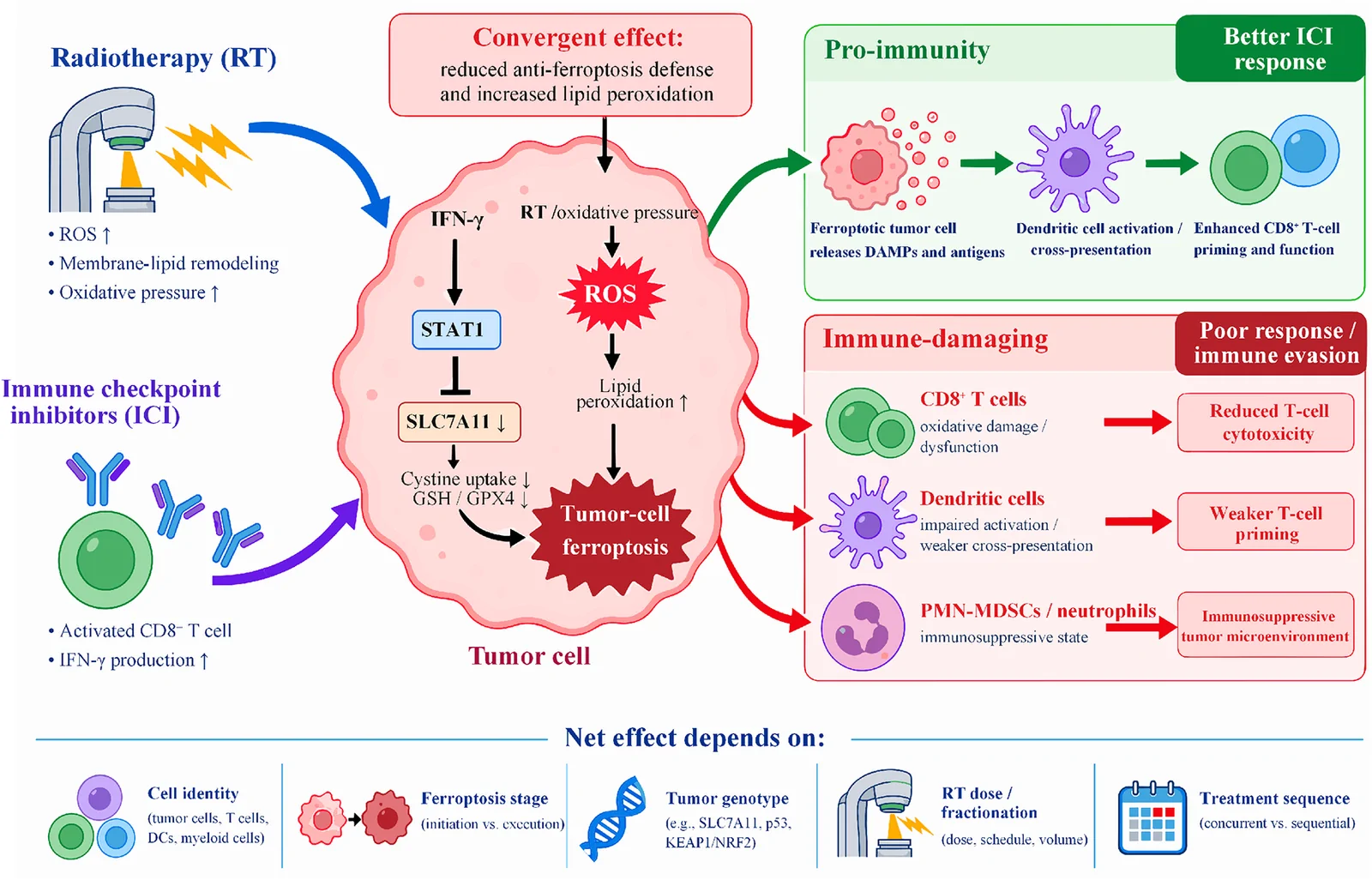
Cell-type-specific effects of ferroptotic pressure during RT and ICI therapy. Radiotherapy (RT) and immune checkpoint inhibitor (ICI) therapy can increase tumor-cell ferroptotic pressure through complementary mechanisms. RT increases reactive oxygen species (ROS), membrane-lipid remodeling, and oxidative stress. ICI-activated CD8^+^ T cells produce interferon-γ (IFN-γ), which activates STAT1 signaling and represses SLC7A11. These signals reduce cystine uptake and glutathione (GSH)-dependent GPX4 activity, increasing lipid peroxidation and tumor-cell ferroptosis. The release of damage-associated molecular patterns (DAMPs) and tumor antigens may support dendritic-cell activation, cross-presentation, and CD8^+^ T-cell priming. These responses may improve ICI responsiveness. Excessive or non-selective oxidative stress can also damage CD8^+^ T cells and impair dendritic-cell function. Ferroptosis in polymorphonuclear myeloid-derived suppressor cells (PMN-MDSCs) and neutrophils can promote immunosuppressive states. The net effect depends on cell identity, death stage, tumor genotype, RT dose and fractionation, and treatment sequence.

Experiments in immune cells provide a basis for these safety considerations. Matsushita et al. showed that GPX4 protects activated T cells from lipid-peroxidation-dependent death and sustains their expansion during immune responses (57). Ma et al. connected CD36-dependent fatty-acid uptake to CD8^+^ T-cell ferroptosis in tumors. Xu et al. linked CD36-mediated uptake of oxidized lipids to lipid peroxidation and reduced effector function (59, 60). The same treatment could therefore eliminate susceptible malignant cells while damaging lymphocytes needed for ICI efficacy. Tumor-volume measurements and bulk-tissue lipid oxidation may obscure this difference. Evaluation of a combination should include antigen-specific T-cell function alongside lethal lipid injury in tumor cells.

## 4 Radiotherapy-induced ferroptotic pressure and adaptive defenses

Ionizing radiation can promote ferroptosis through complementary mechanisms. Water radiolysis increases reactive oxygen species, and radiation expands oxidizable membrane-lipid substrates. ACSL4-dependent phospholipid composition also affects susceptibility. ATM-dependent repression of SLC7A11 reduces cystine uptake and glutathione synthesis, thereby weakening GPX4-mediated lipid-peroxide detoxification (22, 65, 66). Radiation-induced mitophagy and metabolic remodeling can further increase free fatty acids and iron-dependent oxidative stress, whereas NRF2-SLC7A11 activity can confer radioresistance (67, 68). The contribution of ferroptosis varies by tissue. ACSL4-dependent processes can support tumor control and contribute to radiation injury in normal intestine (69).

Direct studies of RT and ferroptosis distinguish lipid injury from DNA-centered radiosensitization. Lei et al. showed that ACSL4 ablation reduced radiation-associated ferroptosis and promoted radioresistance. Inhibition of SLC7A11 or GPX4 sensitized resistant cells (65). Ye et al. found that ferroptosis inducers cooperated with cytoplasmic microbeam irradiation, whereas nuclear irradiation showed no corresponding interaction. The combination effects were associated with lipid peroxidation, with no additional DNA damage or caspase activation (66). These experiments support a contribution of ferroptosis to radiation killing. Assessment of the immune consequences requires models with an intact host immune system. Cell cultures, xenografts, and patient-derived tumor preparations answer complementary questions about tumor-cell sensitivity.

FSP1, mitochondrial DHODH, NRF2-dependent antioxidant programs, and PROM2-associated iron export provide defenses alongside the SLC7A11-GSH-GPX4 axis (42, 44, 48, 49). A 2026 study examined DHODH in lung cancer models that acquired radioresistance through repeated irradiation. RT induced DHODH through CREB-dependent transcription. DHODH supported radioresistance by generating mitochondrial ubiquinol, which suppresses lipid peroxidation, and by maintaining *de novo* pyrimidine synthesis for DNA repair. Pharmacological or genetic inhibition of DHODH cooperated with RT and IFN-γ to increase ferroptosis. The interaction included IFN-γ elicited by anti-PD-1 therapy (70). These experiments identify DHODH as a targetable defense in preclinical combinations of RT and ICI.

RT dose may affect ferroptosis and immunity in different ways. High single doses can produce substantial oxidative injury while also inducing TREX1 and damaging immune or stromal compartments (10). Luo et al. found that low-dose RT, 0.5 Gy *in vitro* and 1 Gy × 5 *in vivo*, enhanced ferroptosis-related readouts with ICI therapy. The response involved the NRF2/HO-1/GPX4 axis, with ferrostatin-1 rescue in experimental systems (71). The activity observed at low doses suggests that the relationship between fractionation and ferroptosis depends on the model and treatment context.

## 5 Coupling local ferroptotic stress to antitumor immunity

Ferroptosis and immune activation intersect through several partially independent routes. ICI-activated CD8^+^ T cells release IFN-γ, which activates STAT1 signaling and represses SLC7A11 in tumor cells. RT activates ATM and also represses SLC7A11. The convergence increases tumor-cell lipid peroxidation. Immune activity can thereby lower the ferroptotic threshold of irradiated tumor cells (21, 22). ACSL4-dependent lipid composition further links CD8^+^ T-cell activity to tumor ferroptosis (72).

The IFN-γ response also depends on lipid substrate availability. Liao et al. linked CD8^+^ T-cell-derived IFN-γ and arachidonic acid to ACSL4-dependent tumor ferroptosis (72). Their findings extend the mechanism beyond cystine deprivation and suggest that immune signaling and membrane composition should be assessed together. A tumor may receive an inflammatory signal yet retain protection through its lipid composition or parallel antioxidant defenses. In an immune-rich biopsy, lipid oxidation may also originate in infiltrating cells. Cell-resolved measurements help separate the intended tumor response from oxidative dysfunction of lymphocytes, particularly when an intervention produces lipid-peroxidation signals without the expected therapeutic response.

RT-generated cytosolic DNA and micronuclei activate cGAS-STING and type I interferon pathways. These responses support dendritic-cell recruitment, cross-presentation, and T-cell priming (73–76). DNA sensing may cooperate with antigen release during ferroptosis. Their coexistence in an RT-induced vaccination response, however, leaves open how much each mechanism contributes to immune control.

The cell-specific and temporal effects described in Section 3.2 also influence interpretation of treatment responses. Studies should identify the cell population undergoing ferroptosis, its stage of death, and the RT-ICI schedule under evaluation (50, 61, 77).

Insufficient tumor-cell ferroptosis may help sustain an immunosuppressive environment after RT. Wu et al. found that radioresistant tumor cells upregulated ferroptosis-resistance genes and released more TGF-β1, promoting regulatory T-cell (Treg) accumulation. Sorafenib with irradiation reduced tumor growth and Treg accumulation in radioresistant models (78). These findings link ferroptosis resistance with Treg-mediated immune suppression and radioresistance. The study contained no ICI arm, so its relevance to ICI resensitization remains indirect.

## 6 Ferroptosis as a context-dependent determinant of ICI responsiveness

The evidence for ferroptosis-dependent ICI responsiveness addresses several distinct treatment settings. In otherwise responsive preclinical systems, increasing tumor-cell ferroptotic pressure can improve the effect of ICI therapy. Lang et al. combined a single 8-Gy dose with CTLA-4 or PD-L1 blockade. The combination increased tumoral lipid peroxidation and tumor control, and liproxstatin-1 weakened the combined effect (22). Nanomedicine and metabolic-intervention studies have extended this approach, although the extent of functional validation differs among them (70, 79, 80).

Ferroptosis-directed sensitization appears most plausible when the immune processes required for ICI therapy remain at least partly intact. Profound T-cell exclusion, defective antigen presentation, or loss of IFN-γ/JAK-STAT responsiveness may restrict the benefit of increased ferroptotic pressure. In these settings, the tumor can remain inaccessible or unresponsive to immune attack despite greater susceptibility to lipid injury.

Resistance models should specify the treatment history and the biological defect under study. JAK1/2 alterations associated with primary or acquired resistance can interrupt interferon-dependent tumor responses. Antigen-presentation defects can prevent recognition despite continued checkpoint blockade (6, 7). These mechanisms differ from resistance to lipid-peroxide accumulation in a tumor that remains recognizable to T cells. An acquired-resistance experiment would first document an ICI response followed by progression. It could then compare continued ICI, RT with ICI, and the full ferroptosis-directed combination in that resistant state. Treatment of an untreated, poorly responsive tumor addresses primary refractoriness or sensitization. Evidence for reversal of acquired resistance requires the preceding response and progression history.

Studies of defined primary-refractory tumors provide evidence for context-specific resensitization. Zhang et al. examined LKB1-deficient lung tumors, a genotype associated with primary ICI resistance. LKB1 deficiency increased ferroptosis tolerance and suppressed STING-related immunity. RT reduced DNMT1, altered the OSGIN1 axis, restored ferroptotic susceptibility, and reactivated STING. RT with anti-PD-1 suppressed these tumors through a ferroptosis-dependent mechanism (82). The results support resensitization in this genetically defined model. Its relevance to individual LKB1-deficient human tumors and other causes of primary resistance awaits clinical evaluation.

Evidence for reversal of acquired ICI resistance remains less developed. The DHODH study included anti-PD-1 treatment in models of acquired radioresistance, without establishing acquired ICI resistance (70). Dou et al. linked HSPB1 suppression, RT, and anti-PD-1 to ferroptosis-related tumor control in hepatocellular carcinoma. Ferroptosis-specific rescue and validation of the resistant state were less complete in that study (81). The current findings support enhancement of ICI responsiveness and resensitization in selected biological settings. Direct testing in models with documented acquired ICI resistance would be needed to extend this conclusion.

## 7 Therapeutic strategies and supporting evidence

Several preclinical studies combine RT, a ferroptosis-directed or ferroptosis-associated intervention, and PD-1/PD-L1 blockade. Their designs address different questions. Some test three-component regimens directly, while others examine endogenous ferroptosis during RT with ICI, target ferroptosis defenses, or alter the immune microenvironment. These categories help distinguish an intervention’s proposed mechanism from the treatment combination actually tested. **Table 2** compares the models, RT schedules, treatment sequences, ferroptosis-directed interventions, ICIs, and efficacy or immune endpoints. It also records the functional validation available for each study.

### 7.1 Direct and representative preclinical evidence

Song et al. combined RT and anti-PD-1 with a glutathione-depleting cinnamaldehyde polymer prodrug carrying all-trans retinoic acid. The platform increased ROS, depleted glutathione, and promoted C11-BODIPY oxidation. Component experiments showed ferrostatin-1-sensitive ferroptosis, and the three-component animal regimen increased cytotoxic T-cell responses and tumor control (79). These findings support the formulation as a ferroptosis-oriented combination. Rescue within the complete animal regimen was not tested in a full factorial design, leaving the proportion of benefit attributable to ferroptosis unresolved.

The Song study used A549 experiments to assess tumor-cell mechanisms and xenograft efficacy, and the LLC/C57BL/6 setting to examine an intact immune response (79). These models provide complementary information. The increase in cytotoxic T cells in LLC tumors cannot be attributed solely to the death mechanism observed in A549 cells. Matched carrier, payload, RT, ICI, and combination controls would help resolve the contributions of a multifunctional formulation. Rescue in the complete immunocompetent regimen could then test the dependence of its additional immune benefit on ferroptosis. Antigen release and other redox effects would remain relevant alternative contributors.

Xu et al. combined a CpG-loaded Fe3O4 antigen-capturing nanoadjuvant with RT and PD-1 blockade in 4T1-Luc breast tumor models. The platform captured antigens released after RT and supported *in situ* vaccination. The combination enhanced antitumor immune responses and controlled irradiated and distant tumors (80). Nontargeted metabolomics identified changes in lipid and amino acid metabolism that the authors interpreted as consistent with ferroptosis. The main article did not report a ferroptosis-specific rescue experiment establishing its contribution to the complete regimen. CpG adjuvanticity, antigen capture, iron-dependent redox effects, and checkpoint blockade may each contribute to tumor control. The findings therefore support combination efficacy, while the causal contribution of ferroptosis remains to be established.

Luo et al. examined endogenous ferroptosis during RT with ICI in lung cancer models. Their measurements included lipid peroxidation, iron, glutathione, and the NRF2/HO-1/GPX4 pathway, with ferrostatin-1 rescue supporting a functional contribution. An exploratory cohort of six patients received low-dose RT with sintilimab-based therapy (71). The clinical observations extend the treatment concept to patients, although ferroptosis was assessed in the experimental models alone. **Table 3** places this cohort alongside other clinical studies of RT and ICI.

**Table 3:** Representative clinical studies of RT and ICI and implications for ferroptosis-directed combinations.

Clinical study	Population and design	RT and ICI schedule	Main clinical findings	Implications for further combination studies
PACIFIC (12, 15)	Unresectable stage III NSCLC; randomized phase III after disease control with chemoradiotherapy	Definitive platinum-based chemoradiotherapy followed by durvalumab	Improved progression-free and overall survival	Consolidation provides a defined sequential setting. Additional treatment would need to preserve the benefit and tolerability of the established regimen.
PEMBRO-RT (13)	Advanced NSCLC; randomized phase II	SBRT, 8 Gy × 3, before pembrolizumab	Higher numerical response rate; the prespecified criterion for meaningful clinical benefit was not met	The pre-ICI irradiation schedule offers a setting for mechanistic sampling. Confirmatory efficacy testing remains necessary.
Theelen pooled analysis (16)	Metastatic NSCLC; pooled analysis of two randomized trials	Pembrolizumab with or without RT; sequential and concurrent schedules in the contributing trials	Higher out-of-field response and improved survival signals	Out-of-field response is relevant to systemic immune effects. Schedule differences and overlap with PEMBRO-RT participants affect interpretation.
KEYNOTE-001 secondary analysis (17)	Advanced NSCLC; retrospective secondary analysis	Prior RT with heterogeneous schedules before pembrolizumab	Prior RT was associated with longer progression-free and overall survival	Prior irradiation is a relevant treatment-history variable. Non-randomized RT exposure limits causal interpretation.
Formenti et al. (14)	Metastatic NSCLC; study of RT with CTLA-4 blockade	Fractionated RT with ipilimumab	Responses in a subset of patients and reported immune correlates	Immune-correlative sampling can complement clinical endpoints when evaluating the contribution of an additional metabolic intervention.
Luo et al. exploratory cohort (71)	Six NSCLC patients with progression after platinum-doublet chemotherapy plus sintilimab; uncontrolled cohort	Low-dose RT, 1.2 Gy × 5, with sintilimab-based therapy	Early clinical activity reported	The post-progression setting and low-dose schedule warrant controlled evaluation. The small cohort provides preliminary information on this treatment context.

ICI, immune checkpoint inhibitor; NSCLC, non-small-cell lung cancer; RT, radiotherapy; SBRT, stereotactic body radiotherapy. The studies inform clinical setting, scheduling, and trial design. None prospectively confirmed ferroptosis in patient samples or tested a prespecified RT, ferroptosis-directed agent, and ICI regimen. Translational implications are interpretations for future study. The pooled analysis includes PEMBRO-RT participants and should not be counted as an independent replication.

Mao et al. tested DHODH inhibition with RT and anti-PD-1 in acquired radioresistant 393P lung tumors. A local 10-Gy dose preceded anti-PD-1, 100 μg per mouse, and leflunomide, 10 mg/kg, or BAY-2402234, 4 mg/kg, on days 3, 6, and 9. Liproxstatin-1 restored tumor growth and suppressed the combination-associated increase in 4-HNE. Genetic Dhodh deletion reproduced the pharmacological effect (70). Pharmacological rescue and genetic confirmation provide direct support for ferroptosis-dependent enhancement of the combination in this radioresistant model. Extension to acquired ICI resistance would require a model with that treatment history.

DHODH inhibition affects both mitochondrial lipid protection and nucleotide supply, complicating attribution of the treatment response. Genetic confirmation and liproxstatin-1 rescue strengthen the mechanistic interpretation of the pharmacological results (70). Paired measurements of lipid oxidation and DNA-repair competence could help determine which activity contributes most at a given exposure. A concentration that alters pyrimidine synthesis may still be insufficient to induce selective tumor ferroptosis. Partner-drug selection also depends on this distinction. A defense induced by RT provides a specific treatment target when experiments show that it limits response. Evidence of such pathway dependence would help justify the choice of an additional inducer.

Zhang et al. provide evidence for genotype-defined ICI resensitization, while Dou et al. describe an HSPB1-centered strategy in hepatocellular carcinoma with less complete causal validation (81, 82). Zhu et al. address a separate therapeutic opportunity through selective targeting and ferroptosis of MDSCs after RT (64). Their work illustrates how myeloid remodeling can complement strategies directed at malignant cells.

### 7.2 Dose, fractionation, and sequence

RT schedules affect tumor redox biology and immune composition. A single ablative exposure, conventional fractionation, and low-dose immunomodulatory RT may produce different patterns of ferroptotic injury. Comparisons across studies are also affected by tumor models, ferroptosis-directed interventions, and ICI timing. The low-dose activity reported by Luo et al. illustrates the difficulty of predicting ferroptosis from dose alone (71). Direct comparisons within the same model would provide a clearer basis for selecting a schedule.

The TREX1 experiments clarify how fractionation can affect immunogenic DNA sensing. Vanpouille-Box et al. found that sufficiently high single doses induced this DNA exonuclease and curtailed cytosolic DNA-driven interferon signaling. Fractionated irradiation preserved immune stimulation under selected conditions (10). The findings concern the models examined and leave tissue-specific lipid-peroxide-dependent death as a separate dose-response question. **Figure 3** presents mechanistic convergence alongside a schematic, non-quantitative relationship between dose and biological effects. Fractionation experiments should account for irradiated volume and immune-cell exposure as well as nominal dose per fraction. These features influence the treatment context in which a particular schedule is evaluated.

**Figure 3:**
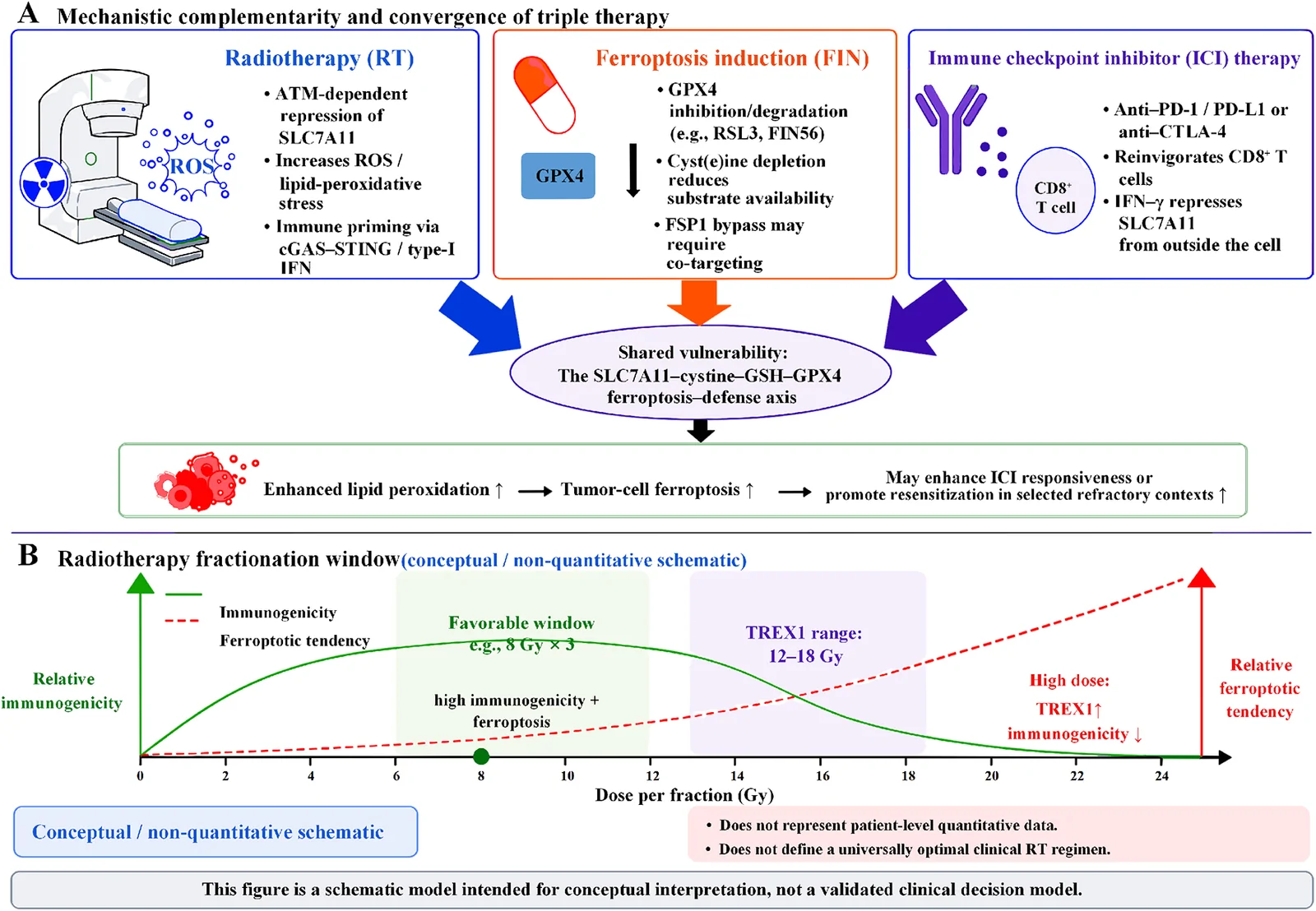
Mechanistic convergence of RT, ferroptosis-directed intervention, and ICI therapy, and the context-dependent influence of RT dose and fractionation. **(A)** Radiotherapy (RT), ferroptosis-directed interventions (FINs), and immune checkpoint inhibitor (ICI) therapy can converge on SLC7A11-dependent cystine uptake and GSH-GPX4 defense. RT increases ROS and lipid-peroxidative stress and can repress SLC7A11 through ATM-dependent signaling. It may also promote immune priming through cGAS-STING and type I interferon signaling. FINs weaken antioxidant defenses through GPX4 inhibition or degradation, cyst(e)ine depletion, or inhibition of parallel protective pathways. ICI therapy reinvigorates antitumor CD8^+^ T cells, whose IFN-γ can further repress tumor-cell SLC7A11. The resulting lipid peroxidation may increase tumor-cell ferroptosis, enhance ICI responsiveness, or support resensitization in selected biologically defined refractory settings. The schematic depicts proposed and experimentally supported interactions; reversal of established acquired ICI resistance requires direct validation. **(B)** The schematic illustrates a proposed relationship between RT dose per fraction, tumor immunogenicity, and ferroptotic pressure. Moderate hypofractionation, exemplified by 8 Gy × 3 in selected preclinical studies of RT and ICI, may allow tumor oxidative stress and immune activation to coexist. At higher single-fraction doses, TREX1 induction and injury to immune or stromal compartments may reduce immunogenicity despite continued oxidative or ferroptotic pressure. The curves and dose ranges are conceptual and model dependent. They have not been derived from quantitative patient dose-response data and do not specify a universal TREX1 threshold or an optimal clinical fractionation regimen.

Treatment sequence may influence both efficacy and injury. A ferroptosis inducer given before RT could increase radiosensitivity while exposing circulating or tumor-infiltrating lymphocytes to lipid-peroxidation stress. ICI treatment after RT may allow antigen release and priming, whereas concurrent treatment may favor convergence of IFN-γ signaling and SLC7A11 repression. The DHODH study administered RT first and began anti-PD-1 with DHODH inhibition on day 3 (70). This schedule offers a tested starting point for comparisons within related models. Studies comparing treatment sequences should document tumor and normal-tissue pharmacokinetics and assess ferroptosis separately in malignant cells and immune subsets.

### 7.3 Clinical experience and implications for combination design

Clinical studies provide practical information about the populations and treatment sequences in which RT and ICI have been evaluated. PACIFIC randomized 713 patients with unresectable stage III NSCLC after disease control with platinum-based chemoradiotherapy. Durvalumab improved median progression-free survival to 16.8 months, compared with 5.6 months with placebo, with a hazard ratio of 0.52. Subsequent analysis showed an overall-survival benefit (12, 15). The trial supports consolidation immunotherapy in this selected population. Its chemotherapy exposure and requirement for disease control before randomization distinguish it from metastatic-disease trials and models of ICI resistance. These eligibility features would matter when considering additional ferroptosis-directed treatment in a similar setting.

PEMBRO-RT evaluated local irradiation before pembrolizumab in 76 randomized patients with advanced NSCLC. RT to one lesion, 8 Gy × 3, yielded a 12-week response rate of 36%, compared with 18% with pembrolizumab alone. The study nevertheless missed its prespecified criterion for meaningful clinical benefit (13). A subsequent pooled analysis of two randomized trials reported higher out-of-field response with RT plus pembrolizumab (16). These observations motivate further evaluation of treatment timing and systemic response. The six-patient low-dose RT cohort provides an additional exploratory setting after progression on platinum-doublet chemotherapy plus sintilimab (71). Its small size and uncontrolled design limit estimates of treatment benefit.

**Table 3** compares the clinical settings, treatment schedules, results, and implications for further study. These trials inform the clinical setting in which an additional ferroptosis-directed intervention might be evaluated. Any such evaluation would need to establish the contribution of that intervention and measure ferroptosis in patient samples. The clinical studies summarized here did not test a prespecified three-component regimen or prospectively confirm patient-level ferroptosis.

## 8 Biomarkers and translational constraints

Biomarkers serve different purposes in this setting. Prognostic markers are associated with outcome regardless of treatment, while ferroptosis-susceptibility markers describe a biological state. Stratification markers define groups for study. Predictive markers identify differential benefit from a particular intervention. High SLC7A11 expression or a ferroptosis-related gene signature may carry prognostic information (83, 84). Their ability to predict benefit from RT, ferroptosis-directed treatment, and ICI requires separate validation. GPX4, ACSL4, FSP1, DHODH, PROM2, oxidized phospholipids, and labile iron have mechanistic relevance, but their predictive value for this combination remains clinically unvalidated.

A biomarker panel could combine tumor genotype with measures of antioxidant defense and lipid susceptibility. Candidate features include LKB1/STK11 and IFN-JAK-STAT status, SLC7A11-GSH-GPX4 and FSP1-CoQ10 activity, and DHODH induction. ACSL4-dependent phospholipid composition and measurements of redox activity or iron in tissue and imaging could add functional context. Cellular localization also matters. The same signal in malignant cells, CD8^+^ T cells, or myeloid cells may have different implications for treatment. Baseline immune-cell states and serial sampling would help distinguish pre-existing susceptibility from ferroptosis induced during therapy.

A validation study could compare pretreatment and on-treatment tissue, resolve malignant, CD8^+^, and myeloid compartments, and relate pathway changes to drug exposure and clinical endpoints. Ex vivo challenge with a defined inducer and rescue could provide a functional measure of susceptibility. Sample handling and culture selection, however, may alter the original tumor state. The SLC7A11 meta-analysis and hepatocellular-carcinoma gene signature describe prognostic associations (83, 84). Predictive validation requires evidence that a marker modifies the relative benefit of an added intervention. A prespecified treatment-by-marker analysis in a comparative trial would address this question more directly than an association within a single treatment group.

Drug maturity and selectivity shape the options for clinical translation (**Table 4**). Erastin-class system xc^-^ inhibitors and direct GPX4 inhibitors remain predominantly research tools because of pharmacokinetic and toxicity limitations. Sulfasalazine and sorafenib are available clinically for other indications, although their pleiotropic effects complicate selective ferroptosis induction and exposure control (85–87). DHODH inhibitors have a more developed pharmacological basis. Their effects on pyrimidine supply and immune cells still impose constraints on dose and scheduling. FSP1-targeting compounds remain preclinical, including compounds that alter FSP1 phase separation to sensitize cells to ferroptosis (88). FIN56 promotes GPX4 degradation and activates squalene synthase, connecting ferroptosis with isoprenoid metabolism (89). This mechanism is distinct from direct FSP1 inhibition. Nanomedicines may improve tumor localization, with additional requirements for manufacturing, biodistribution, and attribution of their biological effects.

**Table 4:** Ferroptosis-directed agents and translational maturity.

Agent/class	Primary target or mode	Development maturity	Evidence with RT-ICI	Key translational constraint
Sulfasalazine	Inhibits system xc^-^ through SLC7A11	Approved for inflammatory disease; repurposing candidate	Mechanistic/preclinical; no validated clinical triad	Pleiotropy, exposure, gastrointestinal toxicity, immune-cell effects
Sorafenib	Multikinase inhibitor with context-dependent system xc^-^/ferroptosis effects	Approved in selected cancers	Indirect; not a selective ferroptosis intervention	Mechanistic ambiguity and off-target kinase effects
Erastin/imidazole ketone erastin	System xc^-^ inhibition	Research/preclinical	No prospective clinical RT-ICI regimen	Pharmacokinetics, solubility, selectivity
RSL3/ML210-class compounds	Direct GPX4 inhibition	Research tools	Mechanistic combination studies	Potential narrow therapeutic window and normal-tissue toxicity
FIN56	GPX4 degradation and squalene-synthase activation	Preclinical research compound	Direct RT combination experiments; not a validated clinical triad	Distinct from FSP1 inhibition; tumor exposure and safety unresolved
DHODH inhibitors: leflunomide, BAY-2402234, brequinar-class	Suppress mitochondrial ferroptosis defense and pyrimidine synthesis	Mixed: approved drug or clinical/preclinical oncology compounds	Strong preclinical RT-anti-PD-1 evidence in radioresistant lung cancer	Pyrimidine depletion, systemic immune effects, schedule and tumor exposure
FSP1 inhibitors	Block CoQ10 regeneration	Preclinical	No clinically validated RT-ICI triad	Potency, selectivity, pharmacokinetics
PDPCA@ATRA	Glutathione depletion, ROS amplification, NRF2-GPX4 modulation	Preclinical nanomedicine	Direct RT-anti-PD-1 triad	Manufacturing, biodistribution, full-regimen causal attribution
CpG-loaded Fe3O4 antigen-capturing nanoadjuvant	Antigen capture, adjuvanticity, iron/redox perturbation	Preclinical nanomedicine	RT + PD-1 blockade with local/distant tumor control	Multifunctionality obscures ferroptosis-specific contribution
HSPB1 targeting	Stress-response/ferroptosis-defense modulation	Genetic preclinical proof of concept	Exploratory RT-anti-PD-1 evidence	No mature selective agent; incomplete rescue-defined validation

RT, radiotherapy; ICI, immune checkpoint inhibitor; GPX4, glutathione peroxidase 4; DHODH, dihydroorotate dehydrogenase; FSP1, ferroptosis suppressor protein 1; CoQ10, coenzyme Q10. Development maturity refers to the status of the agent or class for the indicated ferroptosis-directed use and does not imply regulatory approval for an RT-ICI-ferroptosis combination.

Normal-tissue and immune-cell safety will influence the therapeutic window. Intestinal epithelial cells and activated T cells can be susceptible to ferroptosis (57, 58, 69). A viable regimen needs adequate tumor exposure while preserving antigen-presenting and effector-cell function and limiting additional radiation injury. Cell-selective delivery, transient target inhibition, and spatially restricted activation may help meet these requirements. Their protective effects require experimental assessment alongside antitumor activity.

Unbound drug must reach the irradiated tumor for long enough to engage its target without disabling host immunity. Mouse doses expressed in milligrams per kilogram do not establish achievable concentrations in human tumors. Protein binding, metabolism, penetration, and the timing of irradiation can change both efficacy and toxicity. For nanomedicines, biodistribution studies should assess tumor accumulation together with uptake in myeloid cells and clearance organs. Safety measurements should include T-cell survival and killing, dendritic-cell antigen presentation, and organ-specific radiation injury alongside body weight and histology. Experimental comparisons of tumor and normal-tissue exposure are needed to substantiate claims of selective delivery.

## 9 Discussion

The preclinical evidence links RT, ferroptosis, and ICI response through several complementary observations. These include SLC7A11 repression, functional rescue in combination models, DHODH inhibition in radioresistant tumors, and resensitization of LKB1-deficient tumors (22, 70, 82). These findings support further translational study in defined biological settings. The current evidence is strongest for enhancement of treatment response and selected forms of primary refractoriness. Its extension to acquired ICI resistance requires direct testing.

The effect of ferroptosis depends on its cellular and treatment context. Tumor genotype and parallel antioxidant defenses influence susceptibility, while death stage and the local immune environment affect how damaged cells are recognized. RT schedule and drug distribution alter which populations receive oxidative stress. These factors help explain why tumor-cell ferroptosis can increase local control and antigen availability while lipid injury in CD8^+^ T cells or dendritic cells impairs immunity. Myeloid-cell responses can further redirect this balance. A treatment strategy therefore needs to identify the cells it intends to eliminate and the immune functions it aims to preserve.

### 9.1 Limitations of the present evidence

Most evidence comes from small preclinical studies that differ in tumor type, RT regimen, ferroptosis intervention, and ICI schedule. Multifunctional nanomedicines combine antigen capture, adjuvanticity, ROS generation, and ferroptosis, making their individual contributions difficult to separate. Some reports use expression changes, morphology, or oxidative markers without rescue in the complete combination. The LKB1 and DHODH studies provide detailed mechanistic support within defined genotypes or radioresistant models. Broader application to acquired ICI resistance remains uncertain. The clinical trials, including the exploratory Luo cohort, leave patient-level ferroptosis unconfirmed. The narrative selection process also limits completeness, and the review includes no formal study-quality scoring or meta-analysis.

### 9.2 Future perspectives

Further studies would benefit from immunocompetent models, clinically relevant RT schedules, and factorial treatment arms that separate the contributions of each intervention. Cell-resolved assays and rescue within the complete regimen would improve causal interpretation. Reports should also distinguish untreated primary resistance, adaptive resistance, acquired ICI resistance after response, and acquired radioresistance. Each describes a different treatment history.

Prospective trials could incorporate paired tissue or validated non-invasive measurements to test whether ferroptosis occurs in patients. Associations with benefit would then need mechanistic assessment to determine whether ferroptosis contributes to the response.

## 10 Conclusion

RT and antitumor immunity can converge on tumor-cell ferroptosis through SLC7A11 repression and related metabolic responses. Preclinical studies show improved ICI responses and resensitization in selected, biologically defined refractory tumors. Translation will depend on achieving sufficient tumor exposure while preserving immune and normal-tissue function. Functional rescue, clear definitions of resistance, and cell-resolved biomarkers can guide this work. Clinical trials will need to test the additional value of ferroptosis-directed intervention within a specified RT and ICI schedule, including whether the mechanism contributes to patient benefit. Reversal of established acquired ICI resistance remains a question for direct investigation.
